# *Mycobacterium bovis* Pulmonary Tuberculosis after Ritual Sheep Sacrifice in Tunisia

**DOI:** 10.3201/eid2607.191597

**Published:** 2020-07

**Authors:** Jamal Saad, Sophie Baron, Jean-Christophe Lagier, Michel Drancourt, Phillipe Gautret

**Affiliations:** IHU Méditerranée Infection, Marseille, France (J. Saad);; Aix-Marseille-Université, Marseille (J. Saad, S. Baron, J.-C. Lagier, M. Drancourt, P. Gautret)

**Keywords:** Mycobacterium bovis, tuberculosis and other mycobacteria, sheep, Tunisia, bacteria, ritual sacrifice

## Abstract

A woman in France was diagnosed with pulmonary tuberculosis caused by *Mycobacterium bovis* after a ritual sheep sacrifice in her home country of Tunisia. This investigation sheds light on ritual sacrifice of sheep as a circumstance in which religious tradition and practices can expose millions of Muslims worldwide to this disease.

*Mycobacterium bovis* is historically responsible for zoonotic, deadly tuberculosis and has seemingly reemerged in countries where it had previously vanished following eradication programs in cattle and the pasteurization of dairy products (*1**–**3*). In most cases, *M. bovis* tuberculosis results from consuming unpasteurized milk; extrapulmonary disease is thus the most frequent clinical manifestation (*4**–**6*). Also, *M. bovis* could be an airborne zoonotic pathogen causing pulmonary tuberculosis (*7*). In western Europe countries, most of *M. bovis* human tuberculosis cases are seen in migrants and are associated with travel to the country of origin (*4*,*5*,*8*,*9*). *M. bovis* tuberculosis is traced to animal sources, yet reporting of clinical signs and symptoms is often delayed (*3*). The observation of a woman affected by *M. bovis* tuberculosis who participated in a precisely dated religious practice involving sheep slaughtering provided an opportunity to shed light on these medical aspects.

A 43-year-old unemployed woman born in Tunisia emigrated to France in 2000, married, and had 2 children. The patient had no underlying chronic condition, no medical history, no treatment, no history of smoking, and no toxic habits. She had received the bacillus Calmette-Guérin vaccine during childhood. Her last trip to Tunis and surrounding areas was during July 10–August 28, 2018. The patient denied any contacts with ill persons during her stay. She participated in the Aid-el-Kebir (the Great Festival) Muslim festivities on August 22–23, 2018. After the ritual, her husband slaughtered a veterinary-uncontrolled sheep outside the house by cutting through its neck in an open place and then insufflating air beneath the skin of the dead animal using bellows before butchering the viscera, including lungs, heart, liver, and kidneys, which were put into a container while the digestive tract was put separately into another container. His wife then washed the lungs and the other viscera for ≈2 hours in a confined kitchen, cooked them, and consumed them with her family; she did not experience any injury while butchering the animal. 

After her return to France, the patient was apyretic with productive cough, fatigue, and anorexia, which started exactly 22 days after the Aid-el-Kebir festivities ended. The patient attributed symptoms to allergy and did not consult with a healthcare professional. In December 2018, her respiratory tract symptoms persisted; she also developed fatigue, fever, and night sweats and consulted a general practitioner. In January 2019, a computerized tomodensitometry scan confirmed an abscess in the inferior lobe of the patient’s left lung with thick walls and an infiltrate in the lower lobe of the right lung. The patient reported fever, cough, expectoration, and anorexia, and lost 5 kg within 1 month (body mass index 15); we found crackles in both the left inferior and right superior lobes. She did not report hemoptysis, and her physical examination was otherwise unremarkable. Blood examination showed an iron deficiency in microcytic anemia, and her leukocyte count was normal.

We performed a GenExpert assay (Cepheid, https://www.cepheid.com) and detected *M. tuberculosis* complex DNA in 1 sputum sample; we cultured the *M. tuberculosis* complex CSURQ0209 strain. None of the patient’s other family members had any pulmonary symptoms at any time, and their chest radiographs results were normal. Before the precise *M. bovis* identification was known, we administered to the patient a daily oral regimen of rifampin (480 mg), isoniazid (200 mg), pyrazinamide (1,200 mg), and ethambutol (1,000 mg) for 2 months, followed by rifampin (600 mg/d) and isoniazid (300 mg/d) for 4 additional months, with favorable clinical and radiological outcomes and excellent tolerance. Whole-genome sequence analysis of strain CSURQ0209 (GenBank accession no. PRJEB39431) showed that it grouped more proximately with 1 *M. bovis* strain isolated from a patient from Algeria (Figure).

**Figure:**
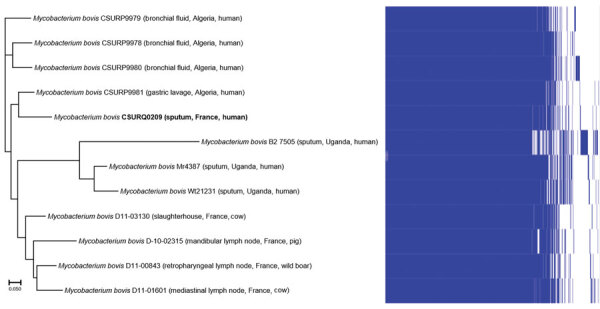
Pangenome tree of *Mycobacterium bovis* from a patient in France (bold; GenBank accession no. CSURQ0209) with 11 reference *Mycobacterium bovis* strains isolated from different regions and hosts. The patient had visited her home country, Tunisia, where she participated in the ritual slaughter of a sheep. Scale bar represents 5% sequence divergence.

In this case, slaughtering a sheep during the annual ritual Aid-el-Kebir festivities was a probable source of infection, although no animal remains were available to confirm this hypothesis. Genome sequence analysis confirmed the identification of *M. bovis*, clustering with isolates from Algeria, in the absence of any other sequence from Tunisia. These results reinforced that this patient had been infected in her native country. During previous years, the festivities took place at the time the family was in France, and no animal sacrifice was performed on these occasions. 

Transmission of *M. bovis* may occur during slaughtering through the inhalation of aerosols exhaled by infected animals (*9*). In this case, the patient was most likely infected by aerosols after prolonged manipulation of the crude viscera. This case potentially concerns millions of Muslim persons worldwide. Our findings indicate that, in countries where ritual animal sacrifices take place, health authorities may want to work with religious authorities to advocate veterinary inspection of slaughtered animals to discard viscera from animals with suspected tuberculosis, in phase with the current World Health Organization roadmap against zoonotic tuberculosis (*10*).
